# Research on coupling control of multiple permanent magnet synchronous motors based on NAISMC and SMDO

**DOI:** 10.1371/journal.pone.0292913

**Published:** 2023-10-20

**Authors:** Yunhui Hao, Ying Zhao

**Affiliations:** School of Intelligent Manufacturing, Changchun Sci-Tech University, Changchun, China; National University of Computer and Emerging Sciences - Lahore Campus, PAKISTAN

## Abstract

The synchronous control system of multi-permanent magnet motor has the characteristics of many parameter variables and mutual coupling. The use of sliding mode control to optimize the parameters in the multi-permanent magnet motor system not only ensures the stability of the system operation, but also improves the control accuracy of the system, which is of great importance in practical applications. Based on this background, the study combines the new adaptive integral sliding mode control (NAISMC) with the improved sliding-mode disturbance observer (SMDO) and uses it for the multi-permanent magnet synchronous motor (MPMSM). In NAISMC, the controller updates and adjusts the parameters of the controller using an adaptive algorithm according to the state of the system and the error signals, which further improves the stability and robustness of the system. SMDO utilizes the principle of the sliding-mode observer to estimate the disturbance of the system, and eliminates the effect of the disturbance on the system by introducing a compensation term. The sliding mode observer calculates the disturbance estimate by comparing the difference between the actual and the estimated outputs. The disturbance estimate is finally used to generate the corresponding compensation signal to eliminate or minimize the effect of the disturbance on the system. NAISMC is combined with SMDO and used in the deviation coupling control of MPMSM. The study established a simulation experiment environment in MATLAB, set the simulation time to 0.4s, and the rated speed of the motor to 1000r/min. The improved sliding mode control scheme is tested, and the results show that the motor output speed, tracking error and electromagnetic torque variation under the improved sliding mode control scheme are smaller than those under the traditional sliding mode control scheme. Under the same simulation conditions, the multi-motor speed synchronization error under the improved sliding mode control scheme is around 0r/min, and its error value is close to 0, so the control effect is higher. In conclusion, the optimization scheme proposed in this study can effectively improve the stability and control accuracy of the multi-motor system.

## I. Introduction

In recent years, traditional car companies have been innovating to address the challenges of high energy consumption and emissions by transitioning to new energy solutions. Multi-permanent magnet synchronous motors (MPMSMs) play a vital role in achieving efficient and environmentally friendly drives in new energy vehicles [1]. By utilizing magnetic field linkage with injected permanent magnet materials, MPMSMs provide consistent magnetic fields across each motor, resulting in higher power factors compared to conventional asynchronous motors and enabling energy savings and environmental benefits [2]. The availability of DC motors, induction motors, and permanent magnetic synchronous machines (PMSMs) currently dominates motor drive systems [3]. PMSMs offer numerous advantages, including low torque pulsation, a wide speed range, simple structure, high torque inertia, and low vibration noise. These features make PMSMs highly versatile and applicable in various new energy drive systems [4]. However, the multi-motor cooperative control of PMSMs poses challenges due to its complex nature as a highly coupled, high-order time-varying nonlinear system. To ensure stable operation, it is crucial to control and optimize the system’s key parameters. Sliding mode control (SMC)is a nonlinear control strategy, which realizes the control of the system state by introducing sliding mode surfaces. Integral sliding mode control is an extension of it, which introduces an integral term on the sliding mode surface to reduce the steady-state error of the system. Its main feature is its adaptive nature, i.e., it can adaptively adjust the parameters of the controller to accommodate the dynamic changes and uncertainties of the system. Sliding mode control, as a nonlinear control strategy, effectively transforms the cooperative control behavior of a multi-motor synchronous control system into a stable linear system, minimizing the impact of external disturbances and improving overall performance control [5].

On this basis, this research focuses on optimizing the design of SMC and sliding mode disturbance observer (SMDO). Firstly, the paper introduces the commonly used multi-motor synchronous control algorithms and types, analyzes the advantages and disadvantages of each type of control method, and then selects the deviation coupling control (DCC) method. To address the limitations of the traditional perturbation observer, the study designs mathematical models of SMDO with improved convergence law. By applying these optimized models to the DCC of MPMMs, it aims to improve their control performance.

The aim of this study is to optimize the synchronous control of MPMSMs by proposing an improved DCC strategy based on the new adaptive integral sliding mode control (NAISMC) and SMDO. The contributions of this research can be summarized as follows. First, the study innovatively optimized the design of the SMC and SMDO. By incorporating the NAISMC and SMDO, the proposed control strategy enhanced the stability and robustness of the MPMSM system, enabling efficient and accurate control of multiple motors. In the next place, through simulation experiments, the effectiveness of the proposed control strategy was validated. The results demonstrated improved stability, reduced tracking errors, and smaller electromagnetic torque fluctuations compared to conventional SMC. The proposed strategy effectively improved the synchronization and control performance of the MPMM system.

While the study primarily focuses on theoretical improvements, it acknowledges the assumptions made regarding ideal operating conditions, accurate parameter measurements, absence of external disturbances, and precise control system implementation. These assumptions provide a foundation for investigating the theoretical effectiveness of the proposed control strategy. However, real-world implementations may require further research and experimentation to address uncertainties and challenges inherent in practical applications. By optimizing the control strategy and demonstrating its improved performance through simulation experiments, this research contributes to the advancement of synchronous control techniques for MPSMSs. The proposed strategy has the potential to enhance the energy efficiency, stability, and overall performance of MPMSM systems, supporting the transition to more efficient and environmentally friendly motor drives in various applications.

## II. Related works

At present, many experts have carried out relevant research on SMC which, as a nonlinear control strategy, is significant in the optimization of various mechanical systems. In view of the low efficiency of the wind energy conversion system of the PMSM, a new adaptive SMC scheme was proposed to optimize it. The adopted SMC method not only retained the robustness of the traditional SMC to external load disturbance and parameter uncertainty, but also reduced the chattering of the system through gain adaptation and design of second-order sliding mode. The proposed design scheme could effectively reduce the steady-state error in traditional SMC and increase the wind energy conversion efficiency of PMSM which can be seen from the experimental simulation outcomes [6]. To optimize the performance of SMC of PMSM, Song J et al. proposed a self-trigger mechanism based on STA algorithm to optimize the SMC of PMSM. By using Lyapunov function, it was proved that the SMC based on STA algorithm could make the tracking error of the system converge to a stable state in a certain time. For external load disturbance, Song J et al. also designed a self-trigger strategy to optimize load disturbance. Finally, a set of nonlinear optimization scheme was designed to adjust the stability of the multi-motor system. The research findings denoted that the proposed self-triggered STA optimization algorithm could effectively improve the performance indicators of the multi-motor system [7]. Cargua-Sagbay D et al. developed a new SMC scheme and applied it to the optimization of flash distillation. It aimed to ensure the stability and robustness of the flash process. The simulation outcomes expressed that the proposed SMC scheme could be optimized according to the characteristics of flash distillation, and overcome some defects of traditional flash distillation in system control [8]. Han L et al. proposed an adaptive neural network non-singular terminal SMC strategy. Its purpose was to settle the issues of path tracking of underwater manipulator due to load interference. The simulation results indicated that the proposed scheme could better track the trajectory of underwater manipulator. It made the whole mechanical system have stronger anti-interference robustness [9]. Zou Q et al. put forward a SMC strategy for PMSM system based on reaching law to resist external unknown load disturbance. The purpose of this method was to enhance the capacity of the system to counteract external unknown load interference by optimizing the multi-motor SMC. Finally, the performance of the optimized SMC was proved through a series of experimental designs [10].

Accurate commutation signals are a prerequisite for efficient operation of brushless DC motors. However, low-pass filters, non-ideal currents, circuit and software suppression can introduce commutation errors, which degrade the performance of brushless DC motors. To eliminate the commutation error quickly and improve the motor performance, a novel compensation method was proposed by Zhao D et al. The compensation time was calculated by a counter, and the start-stop signal of the counter was obtained from the virtual neutral point voltage. Compared with the traditional compensation method based on the integration of the virtual neutral point voltage, this method did not require voltage or current sensors, and the compensation was faster and more accurate [11].Zhou J et al. designed an adaptive fuzzy controller for the tracking control problem of a manipulator. Firstly, based on the introduction of the nominal system controller design, a perfect controller was developed to address the above uncertainties. In this controller, an adaptive fuzzy logic system was used to estimate the uncertainty and eliminate the approximation error by filtering the error, and also compensate for the external disturbances and the uncertainty of the motor drive parameters. Finally Lyapunov stability theory was used to ensure asymptotic convergence of the tracking error. The developed controller was proved to be effective through simulation experiments [12].Chi X et al. investigated the application of proton exchange membrane fuel cells in powered DC motors with a full-bridge converter to realize bi-directional rotation of the motor. In order to accurately regulate the angular velocity and bus voltage of the motor, Chi X et al. proposed an adaptive inverse stepping sliding mode control technique combined with Chebyshev neural network. A control model was formed by constructing an equivalent circuit, and the adaptive control law was obtained by using the Lyapunov function. Finally, this new control technique was compared with the traditional proportional-integral control through numerical simulation, and the results showed that the technique can realize a fast and accurate response in the face of system uncertainty external disturbances [13].A robust nonlinear hybrid control method for separately excited DC motor speed control is presented by R. Afifa et al. Considering that the motor experiences parameter uncertainties and load disturbances in the weak field region, this research is carried out by merging the advantages of adaptive inverse stepping and integral sliding mode control in order to enhance the robustness of the overall system. The results show that the proposed controller can accurately track the reference speed, exhibits robustness, and has good steady-state error accuracy. In addition, the controller proposed in this study exhibits better performance in reducing the stabilization time compared to other controllers [14]. To address the problems of poor fault detection accuracy of permanent magnet synchronous motor drives in speed controllers, A. Khlaief et al. proposed a voltage fault detection technique for power inverters based on signal and sliding mode control. The study used an inverse potential sliding mode observer to estimate the rotor position and stator current and simultaneously diagnose the type of motor control unit system. The results of the study show that the designed fault diagnosis method can effectively detect the types of faults during the driving process of permanent magnet synchronous motors [15].

In summary, MPMSM control is a complex system with many variables, complex parameters, and strong perturbations. During the study of it, external environmental disturbances or internal parameter changes can throw the whole multi-motor control system out of balance. This causes problems such as unstable motor operation and degraded control performance. There have been many studies on the design of SMC methods in multi-motor systems, but there are fewer studies on improved DCC in synchronous control of multi-motors. Most of the optimization schemes for SMC also do not consider the design of SMDO. Although SMC plays a very important role in MPMSM control to provide the best control effect and improve the stability, dynamic performance, accuracy and flexibility of the system, there are many factors affecting each parameter in the multi-motor system, and it is difficult for the individual parameter values to be adaptively adjusted according to the specific environment. To improve this situation, the study optimizes the DCC scheme under MPMSMs. The improved DCC scheme for MPMSM based on NAISMC and SMDO is also proposed by integrating adaptive SMC and disturbance observer. It is aimed at improving the stability and control of the MPMSM system.

## III. Materials and methods

In MPMSM control, DCC can make the coupling between multiple motors tighter and thus improve the overall performance of the system. In addition, the DCC can equalize the load between multiple motors and avoid the problem of overloading one motor. To explicitly represent the coupling relationship between individual motors, the study introduces a comprehensive speed measurement index to optimize the traditional DCC. Based on this, the study further introduces SMC with a disturbance observer to optimize the effect of DCC. Based on the traditional SMC, it is difficult to make the multi-motor synchronous control stable considering that the PMSM is a strongly coupled nonlinear system and therefore responds rapidly to parameter changes and is susceptible to external disturbances. The study uses NAISMC to optimize the conventional SMC. Then, the study uses an improved convergence law to optimize the SMDO, aiming to estimate the total disturbance of the multi-motor system by the optimized disturbance observer, and then to assist in analyzing the coupling control effect of the system.

### A. Study of improved MPMS DCC

At present, algorithms for multi-motor synchronous control (MMSC) emerge endlessly, mainly including PID control, fuzzy control algorithm, neural network algorithm, SMC algorithm, etc. PID control mainly realizes the control of the whole multi-motor system through the design of three types of control parameters: proportion, integral, and derivative. PID control is applied to multi-motor control system. PID control overshoot and other phenomena will occur in the start and stop stage, resulting in large control error of the entire multi-motor control system. Therefore, it is not sufficient for real-time and stability of synchronous control of multi-motor system. Fuzzy control is widely applied in MMSC which is proposed by Zadeh. It mainly regulates its system through fuzzy rules. The capacity of the entire multi-motor system is affected directly by the controller’s design. Because a complex mathematical model is not necessary, it avoids a lot of parameter adjustment. Although there is no need to adjust various parameters, fuzzy control is mainly formulated based on the experience of predecessors, so the formulation of rules will directly affect the system’s control effect. With the development of neural network, neural network control has been used extensively. However, due to its large workload of weight training, there are still some limitations in practical application.

**Fig 1:**
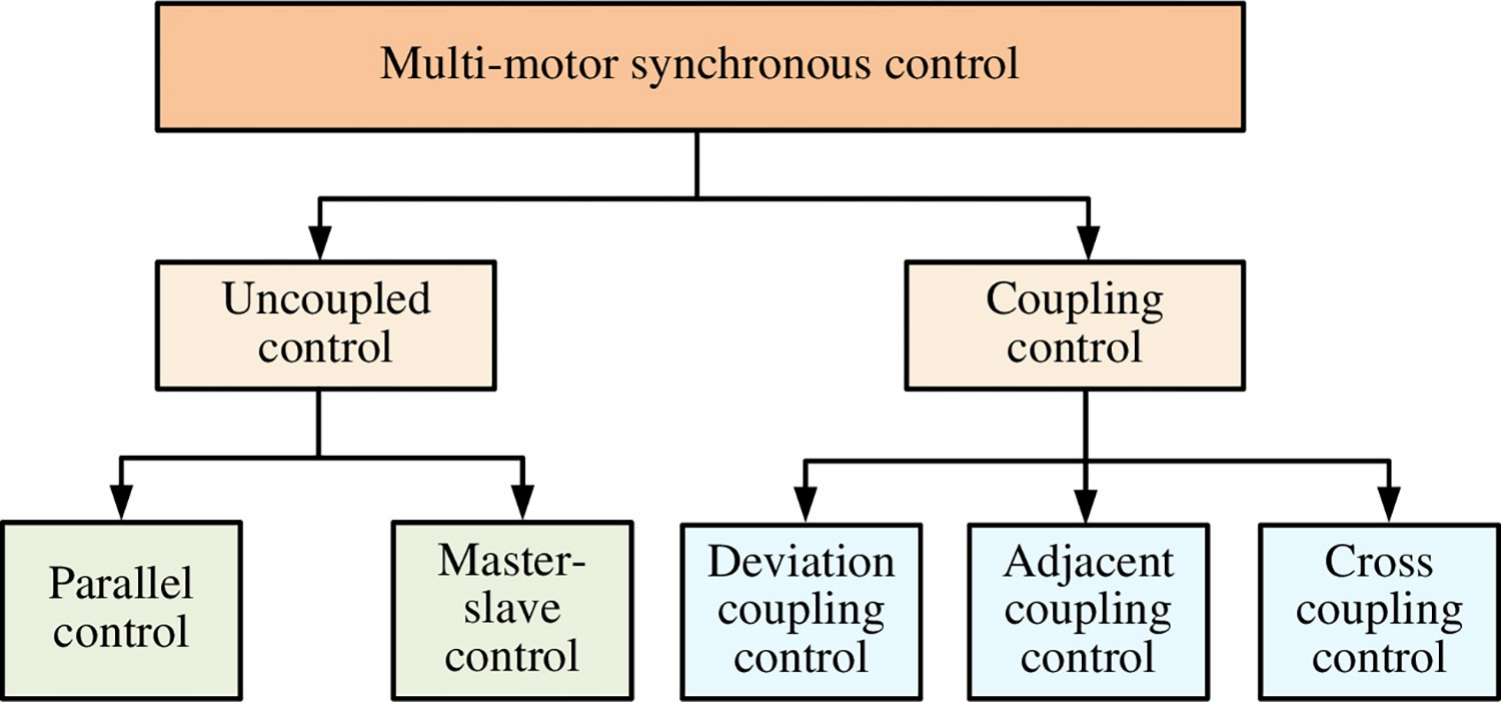
Types of multi-motor synchronized control structure diagram.

Fig 1 shows the type of MMSC. It in a narrow sense means that the speed or motor positions of all motors in the system maintain a certain proportional relationship. In a broader sense, it means that the position or speed of each motor has a certain linear relationship with each other. MMSC is generally divided into uncoupled and coupled control. Uncoupled control mostly has the disadvantages of delayed information transfer and poor information synchronization. The common uncoupled control has two structures: parallel control and master-slave control.

Compared with the above-mentioned control methods, DCC can make the coupling between multiple motors tighter, thus improving the overall performance of the system [16]. It can also equalize the load between multiple motors to avoid the problem of overloading one motor. Each motor can obtain sufficient control signals under DCC to achieve higher precision motion control. Therefore, the study first proposes an improved DCC method to optimize the synchronous control of multiple motors.

**Fig 2:**
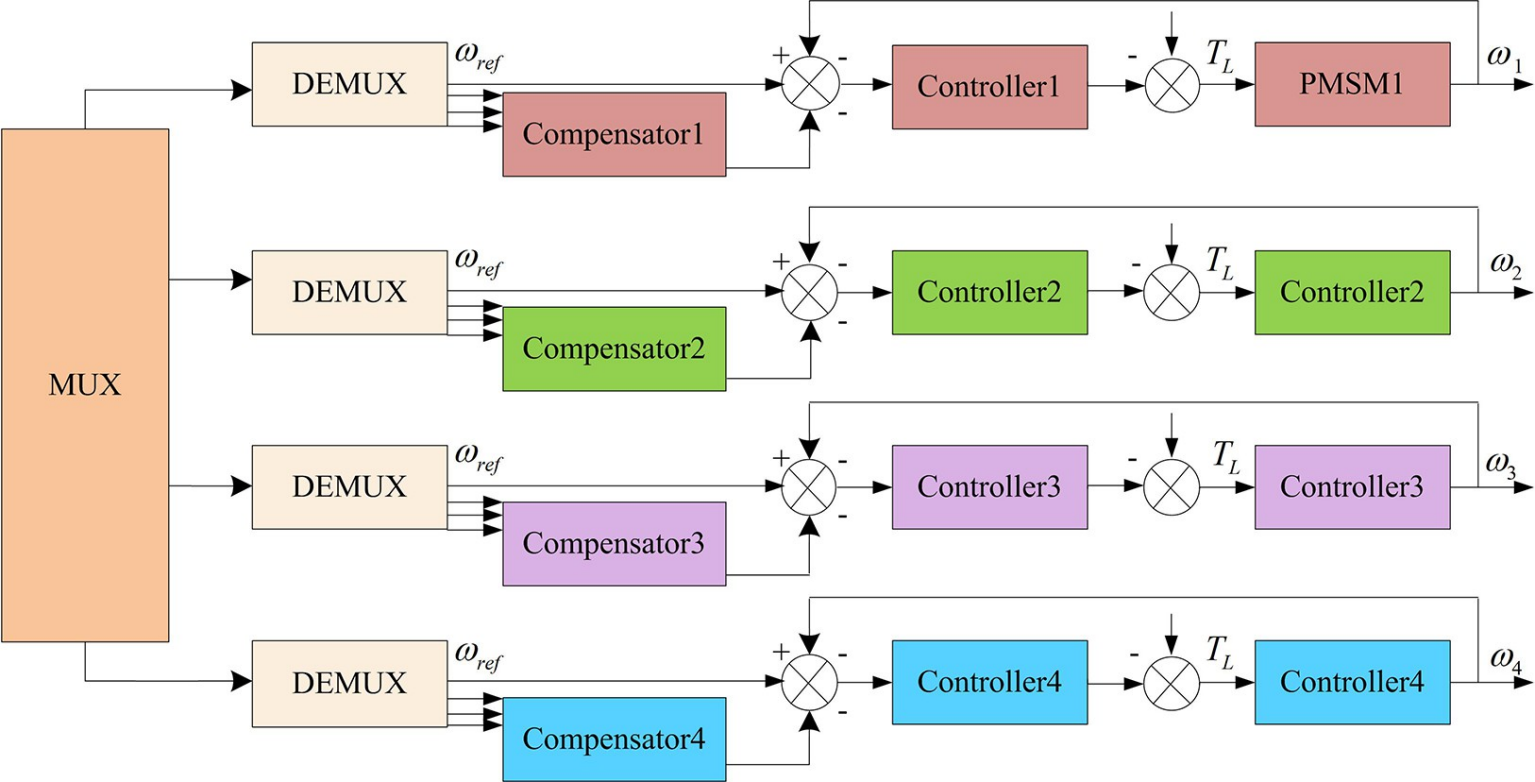
DCC structure diagram.

The basic structure of the DCC proposed by F.J. Perez-Pinal et al. is shown in Fig 2. From Fig 2, the DCC structure mainly consists of a multiplexer (MUX), a demultiplexer (DEMUX), a synchronization compensator (Compensator1~Compensator4), a controller (Controller1~4), and a permanent magnet synchronous motor (PMSM1~PMSM4) are composed.

The basic principle of the DCC is to multiply the speed deviation signal between each motor by the corresponding gain, and then feed it back to the speed signal of each motor. By forming different speed error compensators, it can ensure that each motor can use the speed error for compensation feedback after being disturbed, and then realize MMSC. Taking PMSM1 in Fig 6 as an example, its speed error expression is shown in Formula (1).


$$e_{1}=\omega_{ref}-\omega_{1}$$ (1)

In Formula (1), *e*_1_ means the speed tracking error of the first motor. *ω*_*ref*_ denotes the input speed of PMSM1. *ω*_1_ refers to the output speed of PMSM1. The output speed of the PMSM 1 subtracted from the other motor speeds, multiplied by the corresponding feedback gain compensation coefficient *K*_*ij*_. The values of all the gain compensation coefficients are combined to feed back to the main controller. Thus the design method of PMSM1 synchronous compensator is obtained.


$$K_{ij}=\frac{J_{i}}{J_{j}}$$ (2)

Formula (2) is the calculation of feedback gain compensation coefficient *K*_*ij*_. *J*_*i*_ and *J*_*j*_ represent the rotational inertia of motor *i* and *j* respectively.


$$E_{1}=K_{12}(\omega_{1}-\omega_{2})+K_{13}(\omega_{1}-\omega_{3})+K_{14}(\omega_{1}-\omega_{4})$$ (3)

Formula (3) is the compensation error of PMSM1. Similarly, the compensation error value of other motors can be obtained. *ω*_1_, *ω*_2_, *ω*_3_ and *ω*_4_ respectively mean the output speed of the four motors. *K*_*ij*_ *i*,*j*∈1,2,3,4 stands for different feedback gain compensation coefficients. To clearly express the coupling relationship between each motor, the speed comprehensive evaluation index *E*_1_ is introduced to optimize the DCC [17].


$$\{\begin{array}{c} \omega_{p}=\left(\omega_{\max}+\omega_{\min}\right)/2-{\bar{\omega }}_{m}\\ {\bar{\omega }}_{m}=\sum_{i=0}^{n}\frac{\omega_{i}}{n} \end{array}$$ (4)

In formula (4), *ω*_max_ represents the maximum speed of the controlled motor. *ω*_min_ indicates the minimum speed of the controlled motor. ${\bar{\omega }}_{m}$ means the average speed of all motors.


$$E_{1}^{\prime }=K_{12}(\omega_{1}-\omega_{2})+K_{13}(\omega_{1}-\omega_{3})+K_{14}(\omega_{1}-\omega_{4})+\omega_{p}$$ (5)

Formula (5) is the calculation of PMSM1 compensation error with the introduction of speed comprehensive evaluation index *ω*_*p*_ [18].

**Fig 3:**
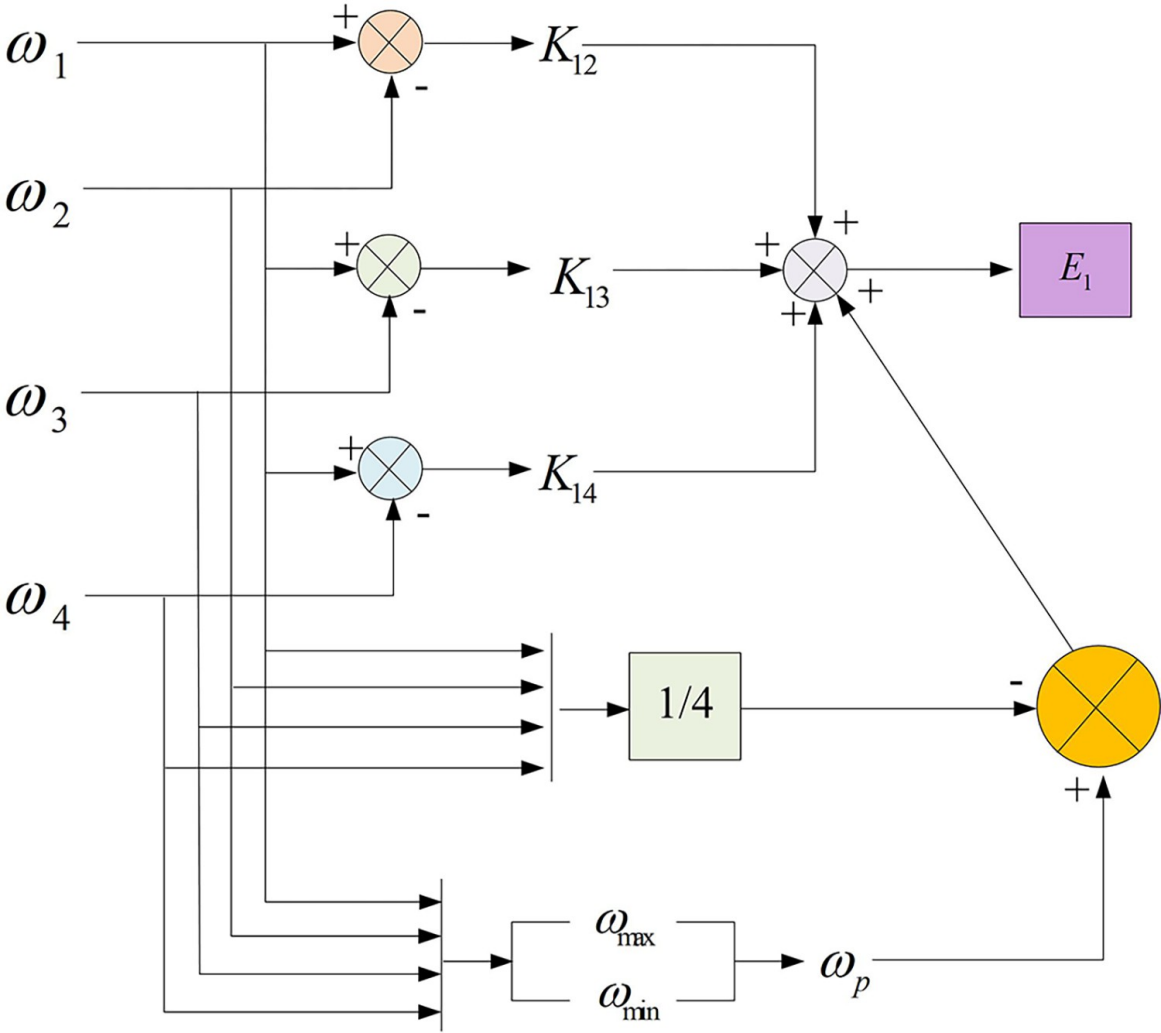
Structure of improved DCC for MPMMS.

The structure diagram of improved DCC is shown in Fig 3. From Fig 3, in the structure diagram of the improved DCC of MPMSMs with the introduction of a comprehensive speed measurement index, the speed of multiple motors can be coupled due to the addition of the measurement index, thus making the speed compensation more accurate and thus improving the coupling control effect. Therefore, the improved DCC is able to better combine the synchronization errors between motors using synchronization error compensators.

Although the improved DCC has a better synchronization error control effect, due to the disadvantages of the multi-motor system itself, such as unstable motor operation and more control parameters, additional SMC and disturbance observer need to be introduced to further optimize the control effect of the system.

### B. Research on coupled control of MPMSMs based on NAISMC and SMDO

In synchronous coupling control, multi-motor system is prone to problems such as unstable operation of the motor system and reduced control performance. In view of this phenomenon, SMC and disturbance observer are used to optimize the whole motor system. SMC is also named variable structure control and its essence is a kind of nonlinear control. In the dynamic process, it can make intentional changes with the current state of the system [19]. It is not affected by object parameters and disturbances, and has the characteristics of fast response, insensitivity to disturbances, and simple implementation.

SMC has a very important role in MPSMS control, which can provide better multi-motor control results. SMC is a sliding mode-based control method that tracks the desired control target by constantly changing the form of the current model. This control method has several advantages. First is to better control stability. By changing the form of the current model, SMC can enhance the stability of MPMSM system. Secondly, it can improve the dynamic performance of the multi-motor system. By switching different forms of sliding mode, the SMC can improve the dynamic performance of the system, so that the system can still maintain the best state under the changing external conditions. Next, it can reduce the system control error. By using sliding mode, SMC can reduce the error and thus improve the accuracy of the system. Finally, flexible control between multiple motors can be achieved. By switching different sliding mode forms, SMC can achieve flexible control effects to meet different drive requirements.


$$s(x,t),\;s\in R^{m}$$ (6)

Formula (6) is the definition of sliding surface function. *S* is the sliding surface function. *x* refers to the system status parameter. *t* indicates the time, and *R*^*m*^ denotes the real number range.


$$\mu_{i}(x,t)=\{\begin{array}{c} \mu^{+}(x,t),s_{i}(x,t)>0\\ \mu^{-}(x,t),s_{i}(x,t)<0 \end{array}\;i=1,2,\cdots ,m$$ (7)

Formula (7) is the solution of SMC function. *μ* is the control variable. *μ*^+^(*x*,*t*)≠*μ*^−^(*x*,*t*) expresses the sliding mode in the sliding state.

Because PMSM is a strongly coupled nonlinear system, it responds quickly to the changes of parameters and is vulnerable to external interference. It is difficult to make the MMSC stable. This paper studies the optimization of traditional SMC by using NAISMC. Taking the surface-mounted PMSM as the research object, its mathematical model expression is shown in Formula (8) [20].


$$\{\begin{array}{c} U_{x}=Ri_{x}+\frac{d\varphi_{x}}{dt}-\omega_{e}\varphi_{y}\\ U_{y}=Ri_{y}+\frac{d\varphi_{y}}{dt}+\omega_{e}\varphi_{x} \end{array}$$ (8)

In Formula (8), *U*_*x*_ and *U*_*y*_ denote the shaft voltage on the *x* and *y* axes respectively. *φ*_*x*_ and *φ*_*y*_ are stator flux chains. *ω*_*e*_ is the electrical angular velocity. *Ri*_*x*_ and *Ri*_*y*_ represent the stator resistance of the motor on the *x* and *y* axes.


$$\{\begin{array}{c} \varphi_{x}=L_{x}i_{x}+\varphi_{f}\\ \varphi_{y}=L_{y}i_{y} \end{array}$$ (9)

Formula (9) is the calculation of stator flux linkage [21, 22]. In Formula (9), *L*_*x*_ and *L*_*y*_ are the inductance components on the *x* and *y* axes, respectively. For the convenience of calculation, the two values should be the same. *φ*_*f*_ is the permanent magnet magnetic chain.


$$J\frac{d\omega_{m}}{dt}=T_{e}-T_{L}-B\omega_{m}$$ (10)

Formula (10) shows the mechanical motion expression of the motor. *J* indicates the moment of inertia. *T*_*e*_ stands for electromagnetic torque. *T*_*L*_ indicates the load torque. *B* means the damping coefficient. *ω*_*m*_ expresses the mechanical angular velocity [23, 24].

The NAISMC is mainly composed of two parts: reaching and maintaining motion. On the basis of rapidly approaching the sliding surface, it is also necessary to maintain the stability of the sliding surface under the action of the control law. According to Formulas (6) to (10), NAISMC is constructed.


$$\dot{s}=-[\frac{k}{a+(1-a)e^{-\delta {\left|s\right|}^{2}}}+\lambda {\left|s\right|}^{\beta }]sat(s)-k_{1}s$$ (11)

Formula (11) shows the mathematical expression of NAISMC [24]. Where, $\dot{s}$ denotes the sliding surface function under the optimization of reaching law. *k* indicates the switching item coefficient, *k*>0. *sat*(*s*) is the introduced saturation function. $\frac{k}{a+(1-a)e^{-\delta {\left|s\right|}^{2}}}+\lambda {\left|s\right|}^{\beta }$ means a toggle item. When |*s*| is large, the switch item becomes $\frac{k}{a}+\lambda {\left|s\right|}^{\beta }$. This can make the whole system converge to stability faster and make the error variable reach the sliding surface quickly. When |*s*| is small, the switching item becomes the coefficient *k*. At this time, the error variables only switch in the boundary layer, and the system is more stable. 0<*a*<1, *λ*>0, *δ*>0, *k*_1_>0, 1<*β*<2. All of the above are motor controller parameters. *e* is a mathematical constant.

At present, there are many researches on the design of disturbance observer, for example, the design of load torque observer which can resist the change of load disturbance by generating electromagnetic torque [25]. The observer can effectively improve the load capacity of the system, but it has the defects of slow response speed, long convergence time, and excessive compensation current. In addition, some scholars have introduced adaptive control algorithm into the disturbance observer to accurately measure the variation of load disturbance. It is compensated to speed loop regulator to reduce load disturbance. The anti-disturbance effect of the observer is better than that of the traditional observer. However, it still has the defects of instability and easy overshoot, so there is still room for optimization. The improved reaching law is used to optimize the SMC. A SMDO based on the improved reaching law is proposed. It aims to optimize the observation indexes of motor load disturbance. For PMSM, due to the limitation of the rated load of the motor, its load effect changes little, so the following expression can be satisfied [26].


$$\frac{dT_{L}}{dt}=0$$ (12)

Formula (12) is the expression for the integration of the load torque. Let the mechanical angular velocity *ω*_*m*_ be the output quantity, *d* be the total perturbation, and *T* be the internal parameter uptake. The mathematical model of the sliding mode perturber is obtained using the integral sliding mode surface as shown in Formula (13)

$$s=e_{\omega }+\lambda_{\omega }\int_{0}^{t}e_{\omega }dt$$ (13)

In Formula (13), *e*_*ω*_ indicates the control law when the speed difference is the input. *λ*_*ω*_ denotes integral sliding mode gain. The approach law algorithm is optimized, so that the final sliding mode perturber meets the requirements as shown in Formula (14).


$$\dot{s}=-qs-h(X)sign(X)$$ (14)

In Formula (14), *X* indicates the independent variable error. *q* is the approach law coefficient. *h*(*X*) meets the limiting conditions shown in Formula (15) [27].


$$h(X)=\{\begin{array}{c} \frac{k_{2}|X|}{1+|X|}\;|X|\leq \eta \\ \frac{k_{2}}{\tau }\;|X|>\eta \end{array}$$ (15)

In Formula (15), *k*_2_ is the approaching law coefficient. *η*, *τ* are the independent variable boundary layer, which satisfies *η*>0 and 0<*τ*<1. Lyapunov function can prove the stability of the disturbance observer.

**Fig 4:**
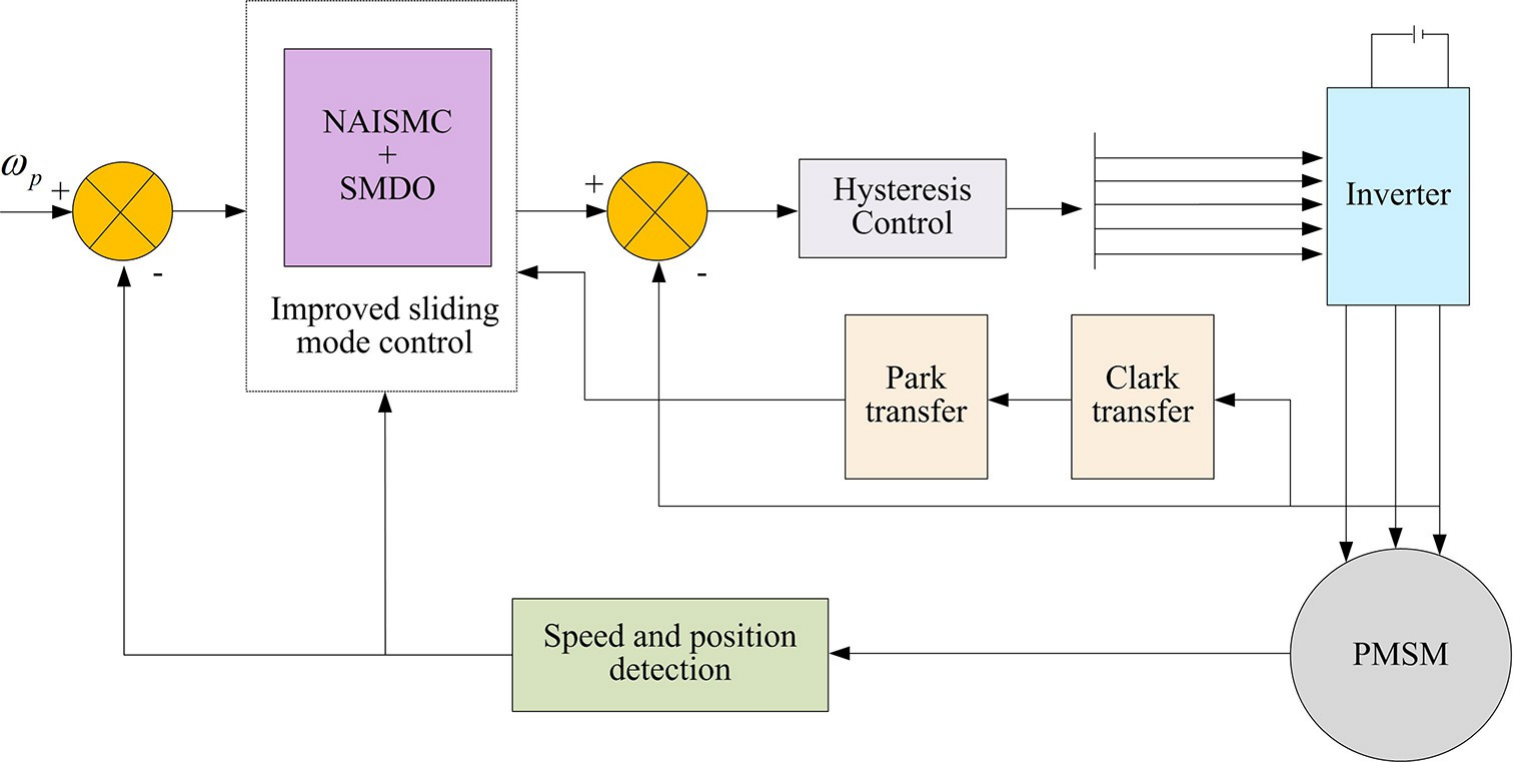
Structure diagram of improved DCC of MPMMS with NAISMC and SMDO.

The improved SMC and SMDO are used in the improved DCC of MPMMS. The control structure schematic diagram is shown in Fig 4. To test the results of improved DCC of MPMMS with NAISMC and SMDO, a simulation experiment will be designed on the basis of this control structure schematic diagram to analyze the motor performance under traditional and improved SMC.

## IV. Simulation experiment

### A. Performance analysis of different motors under different control methods

In order to further test the performance of the motor under various control algorithms, the study utilized MATLAB to build a simulation experimental environment. The hardware and software configuration and parameter settings of the experimental environment are shown in Table 1.

**Table 1:** Table of experimental environment and parameter settings.

Experimental environment	Parameters	Simulation Model Parameters	Value
Computer operating system	Windows 10 64-bit	Resistance/Ω	0.65
CPU	Inter® CoreTM i7-9750H	Inductance/H	0.005
Graphics card	NVIDIA GeForce GTX 1660 Ti 6GB	Simulation time/s	0.4
System Running Memory	16GB	Rated torque/N-m	0
Programming environment	MATLAB	Rated speed/r/min	1000

In Table 1, the software used to run the algorithm for this experiment, the computer hardware configuration, and the ratings of the important parameters of the motor are given. The simulation platform was built under the Matlab experimental environment. The simulation time was 0.4s. The rated speed of motor was 1000r/min. Firstly, the performance of four different PMSM under traditional SMCwas tested.

**Fig 5:**
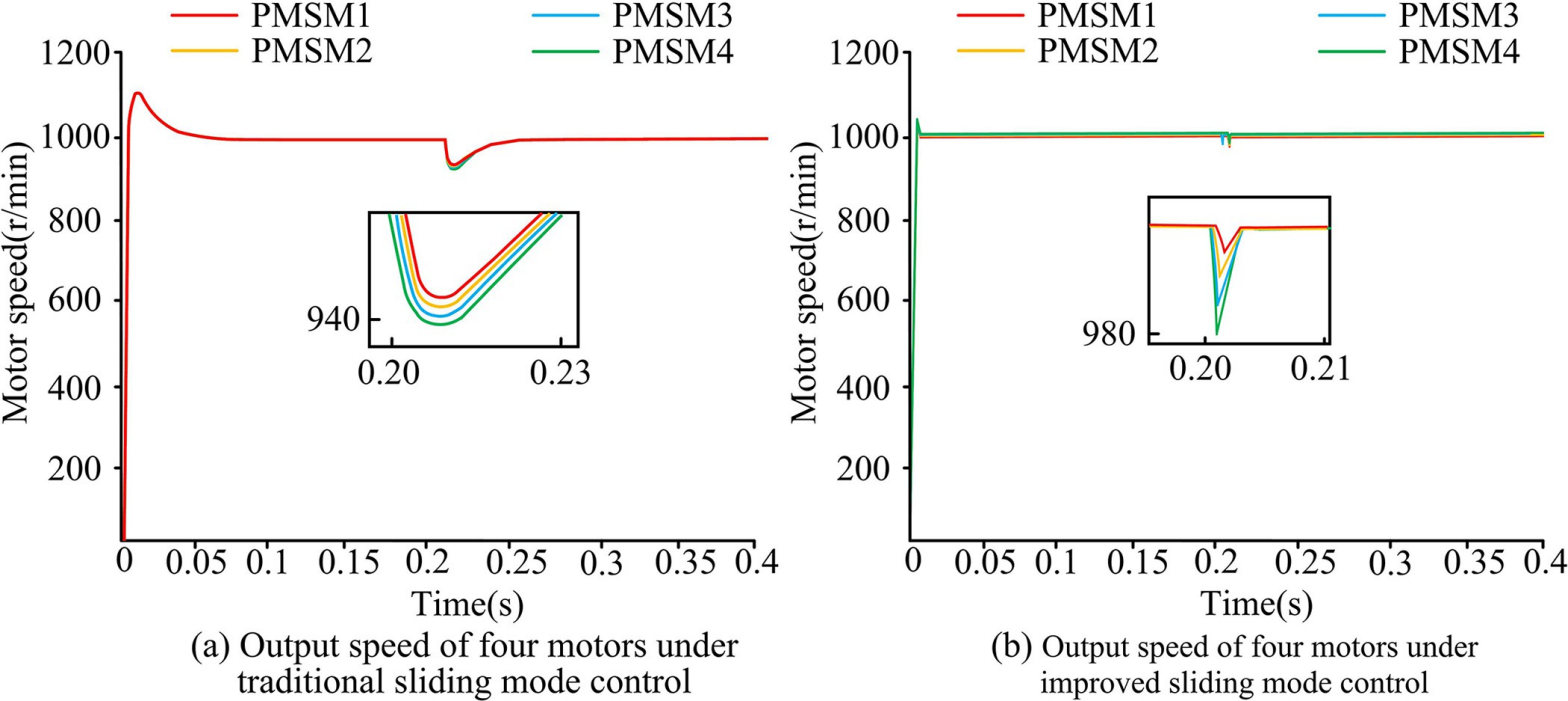
Output speed of four motors under traditional SMC.

The output speeds of the four motors under different SMC are shown in Fig 5. From Fig 5(A), a load torque of 1N-m, 3N-m, 5N-m, and 7N-m was added to the four motors respectively at the simulation time of 0.2s. The time required for the four motors to recover to the target speed was found to be about 0.03. Under the conventional SMC, when the motor was disturbed by external load torque, its rotational performance was affected and it took longer time to recover to the stable speed. From Fig 5(B), when the simulation time was 0.2s, a load torque of 1N-m, 3N-m, 5N-m, and 7N-m was applied to PMSM1, PMSM2, PMSM3, and PMSM4, respectively, and then the rotational speed change of the motor under the improved SMC was observed. The four motors under the improved SMC were able to quickly recover from the varying motor speed to the rated state within 0.01s. This showed that the MPMSM system under the improved SMC was able to perform the speed output more smoothly.

**Fig 6:**
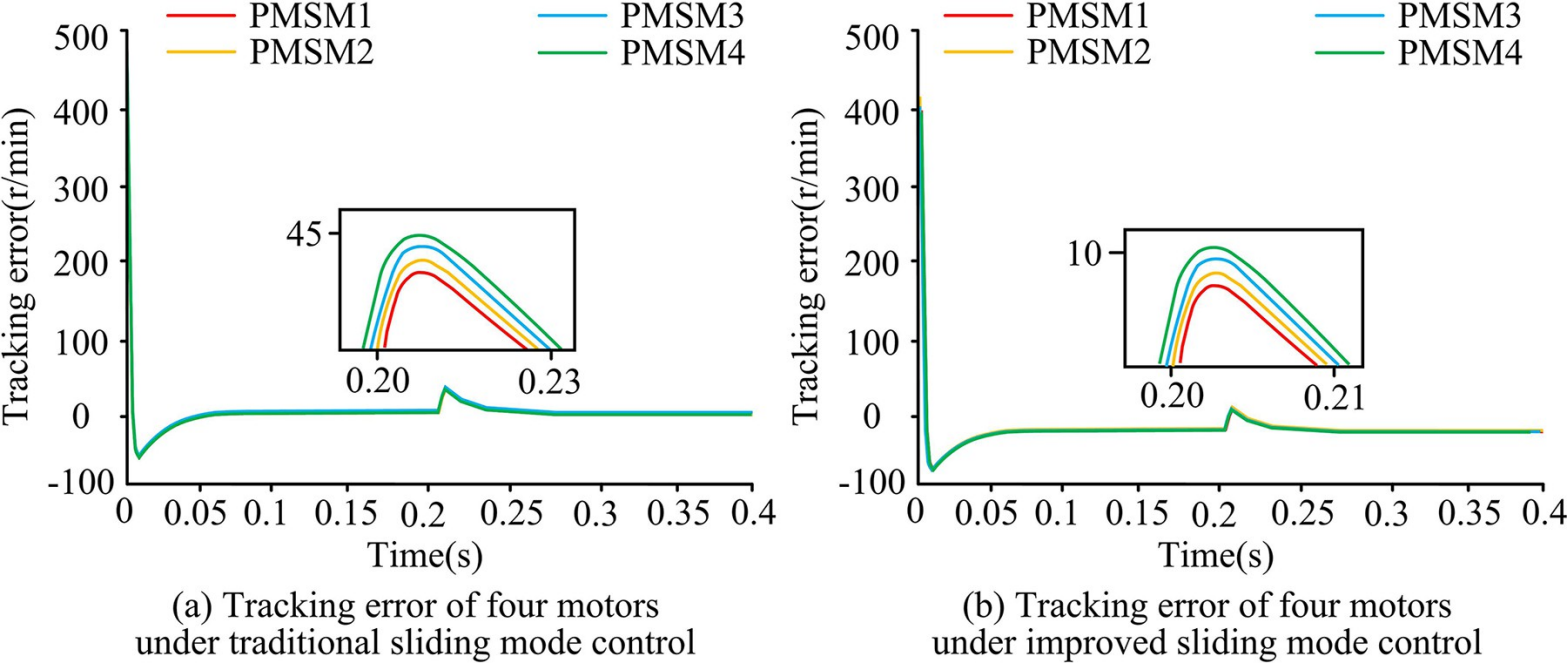
Tracking error of four motors under traditional SMC.

Fig 6 shows the tracking errors of the four motors under different SMC. From Fig 6(A), when a load torque was applied at 0.2s, it would make the tracking speed error of the four motors rise to about 45r/min and gradually return to the stable state after 0.23s.The tracking speed error under the conventional SMC was susceptible to changes in the load torque, which affected the stability of the system. From Fig 6(B), when an external load torque was applied at 0.2s basically, it did not change the tracking speed error value of the four motors. The error range changed briefly from 0r/min to 10r/min and then quickly returned to 0r/min. This was due to the ability of the improved SMC to compensate for disturbances, thus ensuring that the system remains stable in the presence of external disturbances.

**Fig 7:**
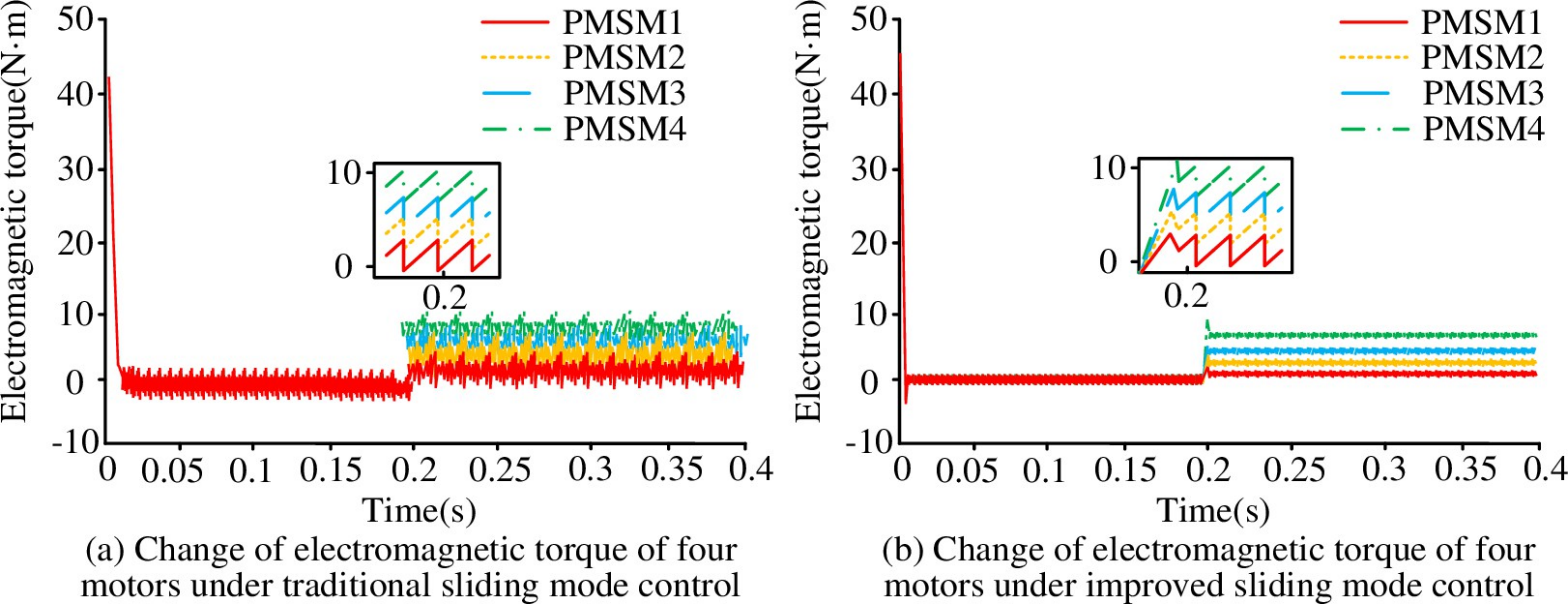
Change of electromagnetic torque of four motors under traditional SMC.

Fig 7 shows the electromagnetic torque variation of the four motors under different SMC. From Fig 7(A), before 0.2s, the electromagnetic torque values of all four motors fluctuate around 0 N-m and the fluctuation range was small. At 0.2s, different sizes of load torque were applied to the four motors. The electromagnetic torque values of all four motors increased, and the fluctuation range of electromagnetic torque waveform was larger. Among them, the electromagnetic torque value of motor 1 increased the least, and eventually fluctuated around 1 N-m. The electromagnetic torque value of motor 4 increased the most, and finally fluctuated around 7 N-m. From Fig 7(B), before 0.2s, the electromagnetic torque values of all four motors were 0N-m and the torque fluctuation range was small. When a different size of load torque was applied to each of the four motors, the electromagnetic torque values of all four motors increased. However, the fluctuation range of the electromagnetic torque waveform was basically unchanged, and it could still remain stable. The final electromagnetic torque value of motor 1was 1 N-m, motor 2was 3 N-m, motor 3 was 5 N-m, and motor 4 was7 N-m. In summary, the motor control performance under the improved SMCwas not easily affected by the external load torque and could still maintain the stability of the whole motor operation.

**Fig 8:**
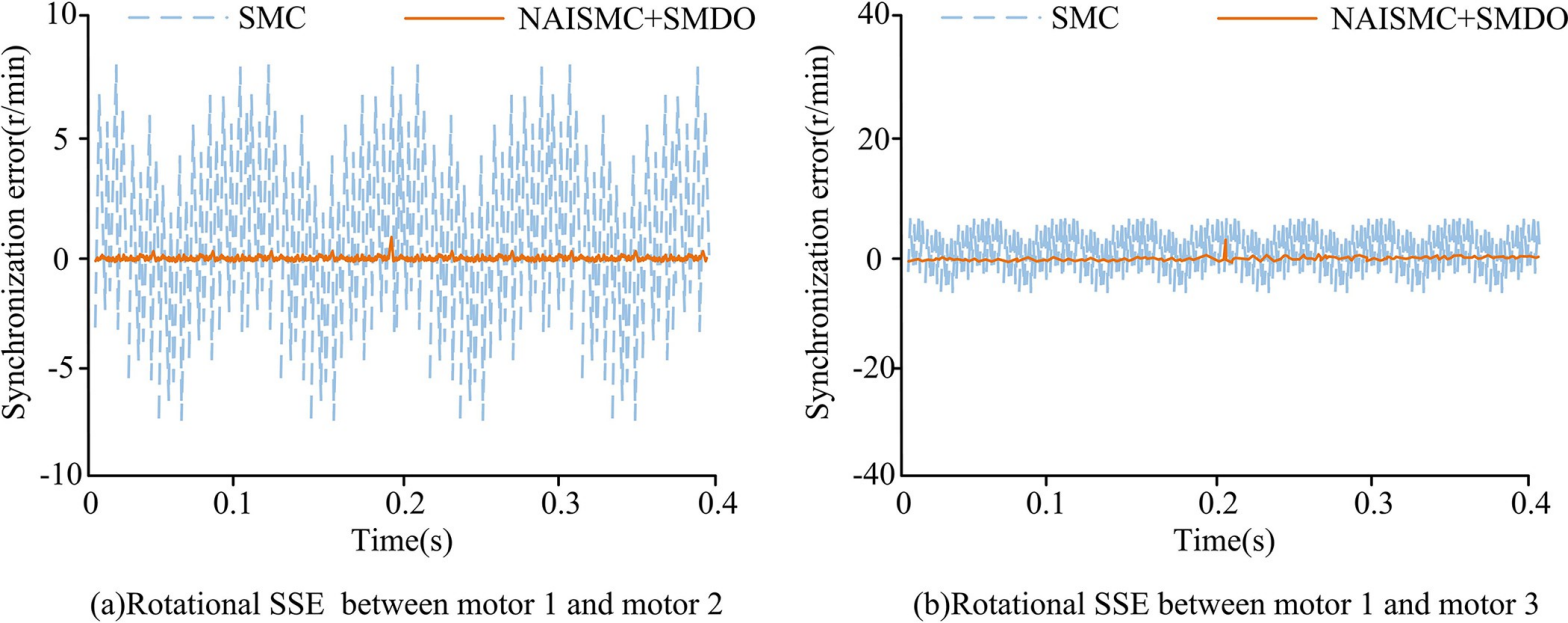
Performance of SSE between motors 1, 2 and 3 under two SMC.

**Fig 9:**
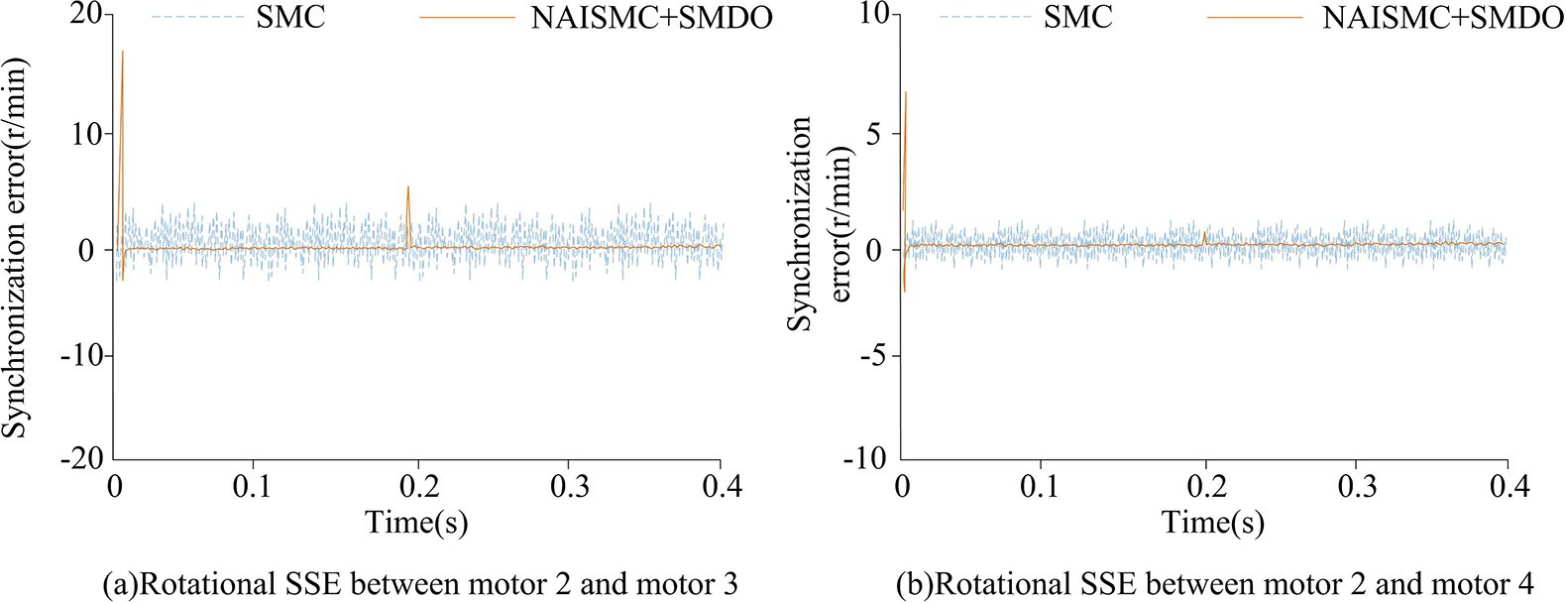
SSE performance of motors 2, 3 and 4 under two SMC.

The speed synchronization errors (SSEs)between different motors under the conventional and improved SMC are shown in Figs 8 and 9. The rated speed of all four motors was set to 3000r/min, the reference speed was set to 1000r/min, and the simulation time was set to 0.4s. When the simulation reached 0.2s, a load torque of 8N-m was applied to each of the four motors. The simulation was completed in the Matlab experimental environment.

Fig 8 shows the performance of SSE between motors 1, 2, and 3 under two SMC. Fig 8(A) shows the performance of SSE between motor 1 and motor 2 under traditional and improved SMC methods. When a load torque of 8N·m was applied at 0.2s, the synchronous error value of the two motors under the improved SMC was increased, but it quickly returned to the original state. During the whole system operation, the motor error fluctuation under the improved SMC was far less than that under the traditional SMC. Therefore, the improved SMC could better guarantee the error stability of different motors during operation. Fig 8(B) shows the performance of SSE between motor 1 and motor 3 under traditional and improved SMC methods. The fluctuation range of SSE between motor 1 and motor 3 was generally smaller than that between motor 1 and motor 2. On the whole, the motor error fluctuation under improved SMC was better. Finally, the synchronous error range of motor 1 and motor 2 under traditional SMC was 7r/min~- 7r/min, and that under improved SMC was about 0r/min. The synchronous error range of motor 1 and motor 3 under traditional SMC was 8 r/min~- 8 r/min and that under improved SMC was still 0 r/min.

Fig 9 shows the performance of SSE between motors 2, 3, and 4 under two SMC. From Fig 9(A),the performance of SSE of motors 2 and 3 under improved SMC was better than that of traditional SMC on the whole. However, when the system just started to run and run to 0.2s, the fluctuation range of synchronous error under improved SMC was large, but both could quickly recover to stable state, and Fig 9(B) was the same. Finally, the SSE range of motors 2 and 3 under traditional SMC was 4r/min - 4r/min, and that under improved SMC was about 0r/min. The SSE range of motors 2 and 4 under traditional SMC is 2r/min - 2r/min, and that under improved SMC was still around 0r/min.

### B. Analysis of the actual control effect of multi-motor under different SMC

Through the above simulation experiments, the motor control performance under conventional SMC was easily affected by the external load torque, thus destroying the stability and coordination of the whole control system. To further demonstrate the effectiveness of the control method used, conventional and modified SMC were applied to the actual multi-motor control to observe the change in motor parameters and the control effect.

Table 2 shows the control stability of each motor and motor system of MPMSM for different control methods. By applying an additional identical load torque to each motor separately, the motor system stability under PID control, parallel control, master-slave control, adjacent cross-coupling control, DCC, SMC, and NAISMC+SMDO+DCC methods were obtained as 75.18%, 77.06%, 77.28%, 80.49%, 85.09%, 87.90%, and 98.67%.The NAISMC+SMDO+DCC method had a better stability of the MPMSM system, which was able to maintain the maximum stability without disturbance in the presence of external load torque.

**Table 2:** Stability of the motor under different control methods.

Control methods	PMSM1	PMSM2	PMSM3	PMSM4	Motor systems
PID control	76.52%	75.23%	73.23%	75.14%	75.18%
Parallel control	77.15%	76.56%	77.18%	76.69%	77.06%
Master-slave control	77.56%	75.65%	76.65%	77.31%	77.28%
Adjacent cross-coupling control	80.56%	79.56%	81.26%	80.22%	80.49%
DCC	85.62%	84.98%	85.17%	84.56%	85.09%
SMC	88.65%	87.69%	87.31%	87.42%	87.90%
NAISMC + SMDO + DCC	98.56%	98.61%	98.45%	98.81%	98.67%

Table 3 shows the time taken by each motor of a MPMSM to recover to normal torque after being disturbed by the load torque for different control methods. By applying an additional identical load torque to each motor, the motor system torque recovery times for the PID control, parallel control, master-slave control, adjacent cross-coupling control, DCC, SMC and NAISMC+SMDO+DCC methods were 12.217s, 8.994s, 8.469s, 5.779s, 5.365s, 3.251s, and 0.012s respectively. This showed that the NAISMC + SMDO + DCC method could recover the steady state more quickly in the presence of external disturbances, which indicated that the system had better control performance and could ensure that the motor was subject to minimal disturbances.

**Table 3:** Recovery time of the motor after disturbance with different control methods.

Control methods	PMSM1	PMSM2	PMSM3	PMSM4	Motor systems
PID control	12.156s	12.265s	11.956s	13.012s	12.217s
Parallel control	8.563s	9.021s	8.697s	9.268s	8.994s
Master-Slave Control	8.659s	8.156s	8.698s	8.348s	8.469s
Adjacent cross-coupling control	5.698s	5.896s	5.745s	5.845s	5.779s
DCC	5.264s	5.114s	5.018s	5.536s	5.365s
SMC	3.265s	3.026s	3.211s	3.329s	3.251s
NAISMC + SMDO + DCC	0.012s	0.011	0.009	0.015s	0.012s

## V. Conclusion

To improve the stability and accuracy of synchronous control of multi-permanent magnet motors, the study optimized the traditional SMC method. An improved DCC strategy based on NAISMC and SMDO for MPMSMs was proposed. Experimental results showed that when different load torques were applied to the motors, the system under conventional SMC took0.03s to recover the steady state. Meanwhile, the tracking error range of the four motors rose to about 45r/min and the electromagnetic torque fluctuations were large. On the contrary, the motor system under the improved SMC took only 0.01s to return to the steady-state output speed, the tracking error rose up to about 10r/min, and the fluctuation amplitude of the electromagnetic torque was also smaller. Under the improved SMC, the synchronization error between two different motors was close to 0r/min. Under the conventional SMC, the synchronization error was much larger than 0r/min. Although the proposed method had good simulation results and practical effects, there were still some errors in practical applications due to the large amount of parameter adjustment, and further improvements are needed.

## VI. Future work

In conclusion, this study has made significant contributions to the field of synchronous control of multi-permanent magnet motors by proposing an improved DCC strategy based on NAISMC and SMDO for MPMSMs. The experimental results have demonstrated improved stability, reduced tracking errors, and smaller electromagnetic torque fluctuations compared to the conventional SMC approach. However, there are still areas that can be further explored and improved:

1. Parameter Optimization and Adaptive Control: The proposed control strategy involves manual parameter adjustment. Future work can focus on developing advanced optimization algorithms or adaptive control techniques to automate the parameter tuning process. This will enhance the control strategy’s adaptability to varying operating conditions and improve its overall performance.
2. Robustness Analysis and External Disturbance Rejection: Conducting a thorough robustness analysis is crucial to evaluate the control strategy’s performance under external disturbances, uncertainties, and parameter variations. Future work can investigate the control system’s robustness and develop strategies to improve its ability to reject disturbances, making it more suitable for real-world applications.
3. Hardware Implementation and Practical Validation: While the simulation results are promising, implementing the proposed control strategy on a physical MPMSM system will provide practical validation of its effectiveness. Future work can involve developing a hardware setup and conducting experiments to evaluate the control strategy’s performance in real-world conditions.
4. Extended Applications and System Integration: Exploring the application of the proposed control strategy to other types of multi-permanent magnet motors or different mechanical systems will broaden its scope of applicability. Future work can investigate its performance in various domains, such as robotics, renewable energy systems, or electric vehicles, and explore opportunities for system integration and optimization.

By addressing these aspects in future work, researchers can further enhance the proposed DCC strategy based on NAISMC and SMDO for MPMSMs, improve its practical applicability, and contribute to the advancement of control techniques for multi-permanent magnet motor systems.
